# Structure and Reaction Mechanism of Basil Eugenol Synthase

**DOI:** 10.1371/journal.pone.0000993

**Published:** 2007-10-03

**Authors:** Gordon V. Louie, Thomas J. Baiga, Marianne E. Bowman, Takao Koeduka, John H. Taylor, Snejina M. Spassova, Eran Pichersky, Joseph P. Noel

**Affiliations:** 1 Howard Hughes Medical Institute, Jack H. Skirball Center for Chemical Biology and Proteomics, The Salk Institute for Biological Studies, La Jolla, California, United States of America; 2 Department of Molecular, Cellular and Developmental Biology, University of Michigan, Ann Arbor, Michigan, United States of America; Vanderbilt University, United States of America

## Abstract

Phenylpropenes, a large group of plant volatile compounds that serve in multiple roles in defense and pollinator attraction, contain a propenyl side chain. Eugenol synthase (EGS) catalyzes the reductive displacement of acetate from the propenyl side chain of the substrate coniferyl acetate to produce the allyl-phenylpropene eugenol. We report here the structure determination of EGS from basil (*Ocimum basilicum*) by protein x-ray crystallography. EGS is structurally related to the short-chain dehydrogenase/reductases (SDRs), and in particular, enzymes in the isoflavone-reductase-like subfamily. The structure of a ternary complex of EGS bound to the cofactor NADP(H) and a mixed competitive inhibitor EMDF ((7S,8S)-ethyl (7,8-methylene)-dihydroferulate) provides a detailed view of the binding interactions within the EGS active site and a starting point for mutagenic examination of the unusual reductive mechanism of EGS. The key interactions between EMDF and the EGS-holoenzyme include stacking of the phenyl ring of EMDF against the cofactor's nicotinamide ring and a water-mediated hydrogen-bonding interaction between the EMDF 4-hydroxy group and the side-chain amino moiety of a conserved lysine residue, Lys132. The C4 carbon of nicotinamide resides immediately adjacent to the site of hydride addition, the C7 carbon of cinnamyl acetate substrates. The inhibitor-bound EGS structure suggests a two-step reaction mechanism involving the formation of a quinone-methide prior to reduction. The formation of this intermediate is promoted by a hydrogen-bonding network that favors deprotonation of the substrate's 4-hydroxyl group and disfavors binding of the acetate moiety, akin to a push-pull catalytic mechanism. Notably, the catalytic involvement in EGS of the conserved Lys132 in preparing the phenolic substrate for quinone methide formation through the proton-relay network appears to be an adaptation of the analogous role in hydrogen bonding played by the equivalent lysine residue in other enzymes of the SDR family.

## Introduction

**Figure 1:**
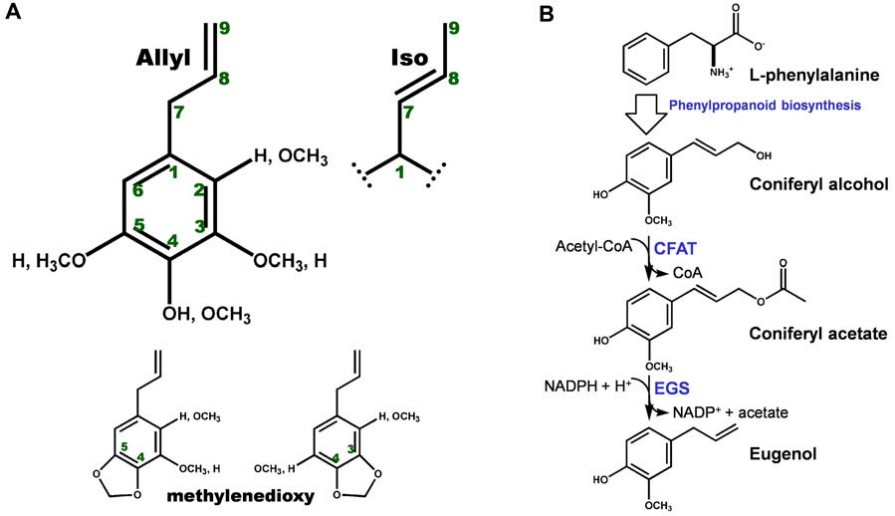
Structure and biosynthesis of the phenylpropenes. (A) Phenylpropene diversity. The basic skeleton of an allyl phenylpropene is shown, along with the numbering of the carbon atoms used in this manuscript. The iso phenylpropenes differ in the position of the double bond (C7 = C8) in the propene side group. For eugenol (an allyl phenylpropene), the phenyl-ring substituents are 2 = H, 3 = OCH_3_, 4 = OH, and 5 = H. The possible methylenedioxy-bridge modifications are also shown. (B) Simplified reaction scheme for the biosynthesis of eugenol from L-phenylalanine and the monolignol alcohol, coniferyl alcohol (CFAT: coniferyl alcohol acetyl transferase; EGS: eugenol synthase).

The phenylpropenes are a diverse group of plant secondary metabolites characterized by a phenyl ring bearing a propenyl side chain (Figure 1A). A variety of phenylpropenes occur in angiosperms, whereas a more limited subset of these compounds exist in gymnosperms. In plants, the phenylpropenes function in defense and interspecies communication. Because some phenylpropenes are toxic to animals and microorganisms, these compounds are typically produced and stored in plant vegetative tissues to act as deterrents against herbivores and microbial pathogens [1]. Moreover, some volatile phenylpropenes are emitted by flowering plants and serve as attractants for insect pollinators [2]. Historically, humans have exploited both the aromatic and toxic properties of the phenylpropenes in perfumes, flavorings, preservatives, and general antiseptics.

The phenylpropenes are derived from coumaryl, coniferyl, and sinapyl alcohol, which are also intermediates in the lignin and lignan biosynthetic pathways. As precursors for phenylpropene production, the monolignol alcohols undergo first acetylation of the C9 hydroxyl group [3] and then reductive cleavage of the acetate moiety to yield the propenyl side group [4], [5]. This reduction reaction is catalyzed by enzymes that produce an allyl propene (with the double bond between C9 and C8) or an “iso” propene (with the double bond between C8 and C7). An example of the former is basil (*Ocimum basilicum*) eugenol synthase (EGS), which converts coniferyl acetate to eugenol, and an example of the latter is petunia (*Petunia hybrida*) isoeugenol synthase (IGS), which converts coniferyl acetate to isoeugenol (Figure 1). Further modifications required for formation of the known natural phenylpropenes include additional hydroxylation of the benzene ring, methylation of any of the hydroxyl groups on the benzene ring, and formation of a methylenedioxy bridge (Figure 1). Some of these modifications may occur before the formation of the propene moiety, but a free hydroxyl group at the *para* position appears to be a requirement for the reduction reaction [4]. The biosynthetic routes to the phenylpropenes generate considerable chemical diversity.

The basil EGS and petunia IGS are closely related to a number of other NADPH-dependent enzymes that act on phenylpropanoid-derived substrates. These enzymes constitute the PIP family, named after the three initially identified members, pinoresinol-lariciresinol reductase (PLR) [6], isoflavone reductase (IFR) [7], and phenylcoumaran benzylic ether reductase (PCBER) [6]. Other enzymes in this family are leucocyanidin reductase [8], and pterocarpan reductase [9]. Notably, several PIP enzymes in addition to EGS and IGS catalyze the reductive cleavage of a carbon-oxygen bond that occurs in a phenyl-ring substituent positioned *para* to the 4-hydroxyl group. The PIP-enzyme catalyzed reductions all involve A-type stereospecificity of hydride transfer from the NAD(P)H cofactor (donation of the nicotinamide C4 pro-R hydride). However, the reaction mechanism of the PIP enzymes remains to be fully characterized. In particular, the cleavage of a carbon-oxygen bond represents an unusual application of a nicotinamide-cofactor dependent reduction, which more typically adds a hydride anion and proton across a double bond in the substrate. The involvement of a quinone-methide (conjugated enone) intermediate in the bond cleavage [4], [9]–[11] is frequently assumed, although solid experimental support has not been reported. Therefore, a direct reductive displacement [4] of the oxygen function by hydride ion cannot be excluded.

We describe here the crystal structure of basil EGS in apo and holo forms, and also as a ternary complex with the cofactor NADP(H) and a designed inhibitor, (7S,8S)-ethyl (7,8-methylene)-dihydroferulate). Previous crystallographic studies of PIP-family enzymes yielded only apo-structures [6], [7], and thus, reliable pictures of cofactor and substrate binding and catalytic mechanism were significantly hampered. Our EGS structures now clearly reveal the interactions formed by the substrate within the enzyme's active site and identify possible catalytic residues. These studies, together with the analysis of the activity of the protein following in vitro mutagenesis of specific residues, provide circumstantial support for a reaction mechanism involving a quinone-methide intermediate and the participation of a key lysine residue in an unusual push-pull like two-step catalytic mechanism.

## Results and Discussion

### Crystallographic structure elucidation for basil EGS complexed with NADP^+^

A structure solution was obtained initially for the orthorhombic crystal form of holo-EGS, which contains two EGS/NADP^+^ complexes per asymmetric unit. A three-dimensional model was refined at 1.7-Å resolution resulting in crystallographic R-factors of 0.244 and 0.267 (working and FreeR, respectively; see Table 1). This refined structure then served as the search model for MR analysis of the monoclinic crystal form. The monoclinic structure also contains two EGS/NADP^+^ complexes per asymmetric unit and was refined at 1.6-Å resolution resulting in crystallographic R-factors of 0.210 and 0.229 (Table 1). For each of the other EGS crystal forms, MR solutions were obtained with either the monoclinic or orthorhombic crystal structure of EGS serving as the search model. A 2-fold rotationally symmetric homodimer is consistently observed as the asymmetric unit in all crystal forms analyzed and solved to date. However, the inter-monomer association within the dimer is not extensive, and in agreement with elution behavior on gel-exclusion chromatography, monomeric EGS is likely the functionally relevant form. In all cases, the entire polypeptide chain of EGS is visible in electron-density maps, except for four residues at the N-terminus, and additionally the NADP^+^ cofactor is extremely well ordered.

**Table 1:** Summary of data collection and refinement statistics for EGS structures

	EGS-NADP^+^ monoclinic	EGS-NADP^+^ orthorhombic	EGS-NADPH	Apo-EGS†	EGS-NADP^+^-EMDF	EGS(Lys132Gln)-EMDF
Space group	*P*2_1_	*P*2_1_2_1_2_1_	*P*2_1_	*P*2_1_2_1_2_1_	*P*2_1_	*P*2_1_
Unit-cell parametersa (Å)	53.8	79.3	54.3	79.4	54.0	53.8
b (Å)	85.9	85.9	86.0	86.3	85.4	86.2
c (Å)	76.2	99.2	76.4	98.2	76.9	76.8
β (°)	107.3	90	107.7	90	107.5	107.6
Monomers per asymmetric unit	2	2	2	2	2	2
Resolution range* (Å)	43-1.60 (1.78-1.60)	100-1.72 (1.84-1.72)	34.-1.60 (1.69-1.60)	43-1.80 (1.88-1.80)	34-1.60 (1.69-1.60)	44-1.80 (1.90-1.80)
Number of reflections measured	316974	492798	268704	443874	354814	167942
Merging R-factor*	0.083 (0.676)	0.110 (0.663)	0.105 (0.286)	0.086 (0.477)	0.129 (0.443)	0.109 (0.341)
Mean (I/σ I)*	9.9 (2.4)	10.0(2.9)	8.9 (1.9)	23.0 (2.5)	8.3 (1.6)	7.9 (1.6)
Completeness*	0. 972 (0.959)	0. 972 (0.961)	0.912 (0.592)	0.956 (0.732)	0.975 (0.950)	0.921 (0.826)
Redundancy*	3.74 (3.74)	6.73 (5.07)	3.3 (2.2)	7.4 (5.1)	4.1 (2.4)	2.9 (2.2)
Number of reflections used	84786	73101	80436	60262	85858	57422
R-factor*	0.205 (0.322)	0.229 (0.361)	0.201 (0.277)	0.224 (0.327)	0.259 (0.318)	0.248 (0.312)
Free R-factor*	0.226 (0.329)	0.256 (0.411)	0.221 (0.306)	0.242 (0.341)	0.286 (0.328)	0.280 (0.361)
Number of amino-acid residues	620	620	620	620	620	620
Number of water molecules	472	547	674	388	374	350
Residues with most favorable conformation (%)	93.0	93.2	93.2	92.1	93.6	92.7
PDB entry	2QW8	2QX7	2R6J	2QYS	2QZZ	2R2G

Merging R-factor = ∑_hkl_ ∑_i_|I_i_(hkl)–〈I(hkl)〉|/∑_hkl_ ∑_i_ I_i_(hkl)

* Values in parentheses describe the highest resolution shell.

† In the apo-EGS crystal structure, a small amount of nicotinamide cofactor is likely present (less than 20% occupancy, as judged from residual electron density at the expected NADP(H) binding site).

### Overall structure of basil EGS and structure comparisons with other IFR-like proteins

**Figure 2:**
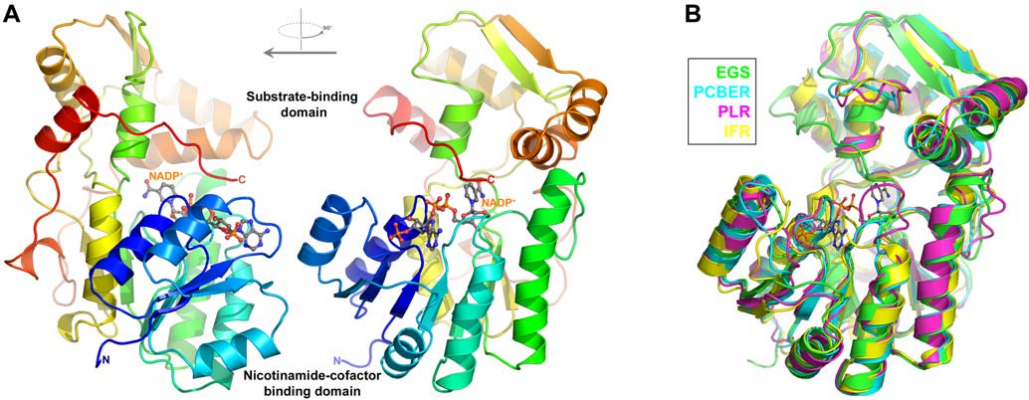
Structure of EGS and comparison with other PIP-family enzymes. (A) Orthogonal views of EGS/NADP^+^ monomer. The polypeptide chain of EGS is represented as a ribbon, with coloring varying from blue for the N-terminus to red for the C-terminus. The atoms of the NADP^+^ cofactor are drawn as balls and sticks, and are colored coded according to element (carbon: gray; nitrogen: blue; oxygen: red; phosphorus: orange). (B) Superposition of the polypeptide-chain backbones of EGS and other PIP-family enzymes (color coding as shown in inset). The NADP^+^ cofactor of EGS is also shown (the structures of the other PIP-family enzymes were determined in the absence of cofactor).

EGS is very similar in polypeptide-chain fold to three other PIP-family proteins that have been structurally characterized, pinoresinol-lariciresinol reductase (PLR) [6], isoflavone reductase (IFR) [7], and PCBER [6]. Structurally, the PIP-family proteins organize around an N-terminal, Rossman-fold domain, containing a core, six-stranded parallel β-sheet flanked on each face by an α-helical layer (Figure 2A). One edge of the core β-sheet provides the extended binding surface for the NADP^+^ cofactor (as discussed further below). The C-terminal polypeptide-chain segment of the PIP-family proteins forms a predominantly α-helical domain, and this C-terminal segment also contributes three additional β-strands to the Rossman-fold domain. The C-terminal domain is presumed (see below) to function in substrate binding. Indeed, this domain together with the last α-helix of the Rossman-fold domain surround a cavity located immediately adjacent to the nicotinamide ring of the NADP^+^ cofactor. Within the IFR-like PIP family, the substrate-binding domains (residues 154–314 in EGS) appear more structurally divergent than the nicotinamide-cofactor binding domains (residues 1–153 in EGS). For example, from comparisons of polypeptide-chain backbones (Figure 2B), EGS differs from PCBER by 1.40 Å (rmsd) overall, but by only 0.83 Å for the Rossman-fold domain alone; and similarly, EGS differs from IFR by 1.63 Å overall and 1.09 Å for the Rossman-fold domain alone. Curiously, PCBER, PLR, and IFR-like EGS-all form 2-fold rotationally symmetric homodimers, but the various homodimeric associations are distinct in each case. An additional structural element unique to EGS (absent in the other PIP-family proteins) is a proline-rich extension at the C-terminus. This tail segment passes across the mouth of the active-site region, and the side chain of the C-terminal phenylalanine residue participates directly in forming the substrate-binding pocket. The positioning of the tail segment in EGS precludes the formation of the homodimeric associations observed in PCBER and IFR.

### Binding of the NADP(H) cofactor to EGS

**Figure 3:**
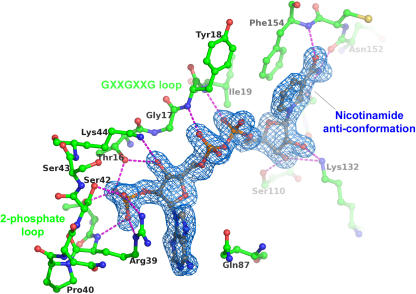
Interactions between EGS and the NADP^+^ cofactor. Only the EGS polypeptide-chain segments that form direct interactions with the NADP^+^ cofactor are shown. Hydrogen-bond interactions formed by the cofactor are represented as magenta dashed lines. Atom coloring is the same as in Figure 2A, except that the carbon atoms of the polypeptide-chain segments are green. The blue-colored contours envelope regions greater than 3σ in the NADP-omit electron-density map.

The structures of EGS complexed with NADP^+^ or NADPH provide the first structural characterization of nicotinamide-cofactor binding by the PIP family of enzymes (previous crystallographic analyses had yielded only apo-enzyme structures). The cofactor is bound through a large number of polar and non-polar interactions (Figure 3). The adenine ring adopts the anti conformation and is sandwiched between the δ-guanido group of Arg39 and the carboxamide group of Gln87. The adenine-ribose lies in the C3′-endo conformation. The ribose ring is packed against the α-carbons of both Gly14 and Gly17, and the central diphosphate group forms hydrogen bonds with the backbone amide-nitrogens of residues 18 and 19. The protein residues involved in these interactions reside within the Gly14-Xaa-Xaa-Gly17-Xaa-Xaa-Gly20 segment, a canonical sequence-motif for NAD(P) binding [12]. The 2′-phosphate is sequestered by a short loop formed by residues 38–42, and is hydrogen bonded to the side chains of Thr38, Arg39 and Ser42. Thr16 from the glycine-rich loop also hydrogen bonds the 2′-phosphate, as well as the adjacent 3′-hydroxyl group. The nicotinamide-ribose has the C2′-endo conformation, and its hydroxyl groups are involved in hydrogen bonds with residue 111′s carbonyl oxygen, Ser110′s side chain hydroxyl moiety, and the side-chain amino group of Lys132. The nicotinamide ring adopts the anti conformation with its B-face stacked against the side chain of Phe154 and its A-face directed towards the substrate-binding pocket. The nicotinamide carboxamide group forms hydrogen bonds to three backbone atoms (ON7 to 154 N, and NN7 to 112 O and 152 O).

Structural differences between the NADP^+^ and NADPH complexes of EGS are small, and center around the nicotinamide ring of the cofactor. The nicotinamide forms a planar group in the oxidized state, but is distorted slightly to a more boat-like conformation in the reduced state. From a comparison of apo-EGS and the EGS-NADP(H) complexes, it also appears that little structural perturbation results from binding of NADP(H), aside from better ordering of the polypeptide-chain segments that form the cofactor-binding cleft.

### Structural comparison of EGS with UDP-galactose 4-epimerase

**Figure 4:**
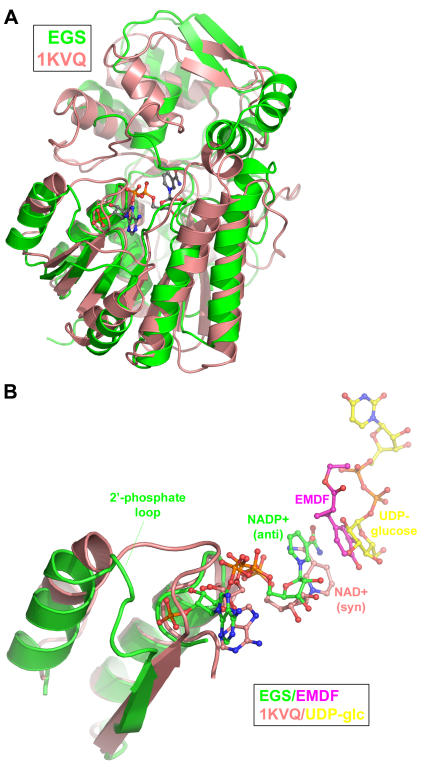
Structural comparison of EGS and UDP-galactose epimerase. (A) Superposition of polypeptide-chain backbones of EGS and UDP-galactose epimerase (color coding as shown in inset). For clarity, only the NADP^+^ cofactor of EGS is shown. (B) Comparison of NAD(P)-cofactor conformation and substrate-analog binding in EGS and UDP-galactose epimerase. The binding of the EGS competitive inhibitor (7S,8S)-ethyl (7,8-methylene)-dihydroferulate (EMDF) is described in detail in the text and Figure 5. The coloring of the polypeptide-chain segments is the same as in (A). The inset shows the coloring used for the carbon atoms of the nicotinamide cofactors, EMDF bound to EGS, and UDP-glucose bound to UDP-galactose-4-epimerase.

The PIP-family enzymes belong to a larger superfamily of NAD(P)-dependent dehydrogenases, the short-chain dehydrogenases/reductases (SDRs) [13]. The most similar member of the larger superfamily is UDP-galactose 4-epimerase [14] (PDB entry 1KVQ), which provided a template for the binding modes of both the nicotinamide cofactor and substrate in the earlier structural analyses of the apo-forms of IFR, PCBER and PLR [6], [7]. Indeed, UDP-galactose 4-epimerase possesses a C-terminal domain that is similar topologically to the C-terminal domains of the PIP-family proteins (Figure 4A). In the UDP-galactose 4-epimerase crystal structure, a cavity within the C-terminal domain is positioned next to the nicotinamide ring of the NAD^+^ cofactor and is occupied by the substrate, UDP galactose. The corresponding cavity in EGS is much smaller in volume and the side chains lining the cavity are more non-polar in character. These properties of the EGS substrate-binding pocket are consistent with the smaller size and greater hydrophobicity of the EGS substrate, the acetate ester of coniferyl alcohol. Notably, the conformation of NAD^+^ bound to UDP-galactose 4-epimerase differs markedly from that of NADP^+^ bound to EGS, particularly in the conformation of the nicotinamide ring (Figure 4B). In the UDP-galactose 4-epimerase/NAD^+^ complex, the nicotinamide ring adopts the syn conformation, consistent with the class-B oxidoreductase activity of the enzyme. In contrast, the anti-conformer of the nicotinamide ring observed in EGS is consistent with the class-A reductase (donation of the pro-R hydride) activity of the PIP-family enzymes. The orientation of the nicotinamide ring in EGS appears to be influenced largely by interactions of the carboxamide group with the polypeptide-chain backbone, an observation also made previously for the SDRs [15]. Also, EGS possesses an additional loop (residues 38–42) that forms a binding pocket for the 2′-phosphate group of NADP(H). This loop is absent in UDP-galactose 4-epimerase, but occurs with variable length in all PIP-family proteins.

### Binding of the EGS-inhibitor EMDF

**Figure 5:**
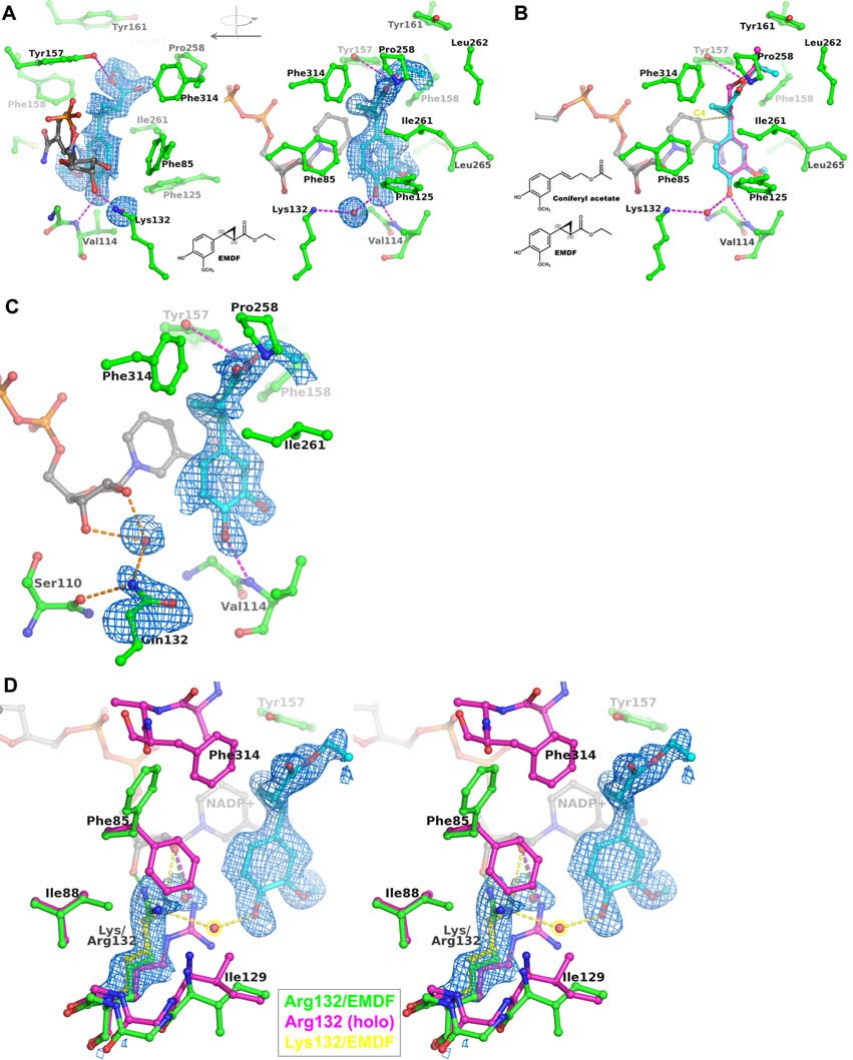
Binding of the competitive inhibitor EMDF by EGS. (A) Orthogonal views of the 7S,8S- stereoisomer of the competitive inhibitor EMDF bound to EGS. Hydrogen-bond interactions formed by the EMDF molecule (cyan colored carbons) are represented as magenta dashed lines. The blue-colored contours envelope regions greater than 2.5σ in the initial F_obs_-F_calc_ electron-density map. The direction of view used in the right panel (approximately perpendicular to the plane of the nicotinamide ring) is maintained roughly in figures 5B–D and 6B. The chemical structure of EMDF is shown in the inset. (B) Modeled binding of coniferyl acetate to EGS. The atom coloring is the same as in (A), with magenta carbon atoms for the coniferyl acetate. The chemical structures of coniferyl acetate and EMDF are compared in the inset. The close interaction between the EMDF C7-atom and the hydride donor of the nicotinamide (C4) is shown as a yellow dashed line. (C) Binding of EMDF to the Lys132Gln variant of EGS. Hydrogen-bond interactions formed by the EMDF molecule (cyan colored carbons) are represented as magenta dashed lines. Hydrogen bonds involving the side chain of Gln132 are shown as orange dashed lines. The blue-colored contours envelope regions greater than 2.0σ in the initial F_obs_-F_calc_ electron-density map. (D) Binding of EMDF to the Lys132Arg variant of EGS (stereo representation). The blue-colored contours envelope regions greater than 2σ in the initial F_obs_-F_calc_ electron-density map for the EGS-Arg132/EMDF complex (green). The altered positioning of the Arg132 side-chain and neighboring residues (most notably Phe85, Ile88, and Ile129) and the disordering of the C-terminal tail (residues 310–314) are apparent with respect to the holo-EGS-Arg132 structure (magenta). For comparison, the position of the wild-type Lys132 side chain and the key bridging water molecule shown in Figure 5A are also shown (yellow).

The co-crystal structure of EGS complexed with a specifically designed inhibitor, EMDF ((7S,8S)-ethyl (7,8-methylene)-dihydroferulate) provides a view of the substrate-binding mode within the active site of EGS (Figures 4B and 5A). This inhibitor is a close structural analog of coniferyl acetate, and carries the same functional groups on the C3 and C4 (*para*) positions of the phenyl (guaiacol) ring. However, EMDF cannot serve as a substrate of EGS because within the C1 substituent, a cyclopropyl group replaces of the C7-C8 double bond and the orientation of the ester is reversed. Our measurements indicate that EMDF acts as a competitive inhibitor of EGS, with an inhibition constant (K_i_ = 0.8 mM) similar to the K_m_ for coniferyl acetate, 0.57 mM. A key interaction between EMDF and EGS is the packing of the inhibitor's phenyl ring parallel to the A-face of the cofactor's nicotinamide ring (interplanar separation 3.4 Å). Notably, stacking of a substrate aromatic-ring against the NAD(P) nicotinamide ring is a common feature of the binding modes of SDRs [16]. In EGS, the nicotinamide ring is, in turn, stacked against the side chain of Phe154. The *para*-hydroxy group of the inhibitor's guaiacol moiety hydrogen bonds with the backbone amide-nitrogen of Val114 and also interacts via a bridging water molecule with the side-chain amino group of Lys132. The residues lining the binding pocket are otherwise predominantly aromatic (Phe85, Phe125, Tyr157, Phe158, Tyr161, and Phe314) and aliphatic (Val114, Ile261, Leu262, and Leu265). In the known EGS and IGS sequences, Lys132, Tyr157, Phe158, and Ile261 are invariant, whereas conservative amino-acid substitutions are observed at the other binding-pocket residues. The inhibitor's 3-hydroxymethyl group is accommodated within a small, non-polar pocket. The observed orientation of the guaiacol moiety would be clearly favored over the reverse orientation (resulting from a 180° rotation around the C7-C1 bond), which would position the hydroxymethyl group in close contact with the nicotinamide ribose and the side chain of Phe85. However, the absence of specific interactions formed by the hydroxymethyl group within its binding pocket is perhaps consistent with the limited ability of EGS to utilize as substrate coumaryl acetate (unpublished data), which lacks a substituent at the 3-position. In addition, the acetate ester of sinapyl alcohol, which bears hydroxymethyl groups at both the 3- and 5-positions, would be expected to be incapable of binding to EGS. The inhibitor's C1 substituent bearing the cyclopropyl and ethyl-ester moieties projects into a cavity formed by the C-terminal domain of the protein. This cavity is capped by the side chains of Tyr157, Tyr161, Pro258, Leu262 and Phe314. With the exception of the C-terminal Phe314, these capping residues form a relatively rigid cage, as indicated by their invariant positioning in all EGS crystal structures and low crystallographic temperature factors. The capping region appears to lack sufficient volume to accommodate a C1 substituent larger than an acetate-esterified propenol. This finding is in agreement with the observed inactivity of EGS toward other esters of coniferyl alcohol, for example coniferyl coumarate [4], which bears a much bulkier substituent.

Curiously, at the inhibitor-binding site described above, some residual electron density is invariably observed, even with crystal samples prepared with EGS protein that had not been purposely exposed to a potential ligand. This density might be due to low-occupancy binding of a small, eugenol-resembling compound that originated from bacterial growth-media derived from yeast extracts. However, soaking experiments of EGS crystals with coniferyl acetate, or other substrate analogs (e.g. coumaryl acetate and 4-bromo-cinnamyl acetate) failed to produce stable complexes with EGS. The difficulty in obtaining EGS complexes with these compounds is possibly explained by the implications of the finding that binding of the true substrate, coniferyl acetate, cannot be readily modeled based on the observed positioning of the EMDF inhibitor compound. The determinative structural feature of coniferyl acetate is the planarity of the propene moiety (C7-C8-C9), which results in steric clashes between the terminal acetate moiety and neighboring residues in the active-site capping region, in particular Tyr157, Ile261 and Phe314. In contrast, the cyclopropyl group of EMDF induces a distinct kink in the conformation of the side group, which steers the end of the side group into a hole formed within the capping region (Figure 5B). The stringent binding selectivity of EGS is further emphasized by the observation that only the 7-(S),8-(S) stereoisomer of EMDF complexed with EGS; the 7-(R),8-(R) isomer (present at low levels in the EMDF preparation) was excluded, due to poorer steric complementarity with the EGS active site.

### 3D structure determination and in vitro mutagenesis suggests a reaction mechanism for EGS

Although the binding of EMDF exploits shape features of the EGS active site that are inaccessible to the coniferyl acetate substrate, the structure of the EGS-NADP^+^-EMDF complex nevertheless provides a useful framework for probing the EGS enzymatic mechanism. Together with the observed effects on catalytic activity of specific amino-acid replacements (Table 2), as described below, the structure provides compelling support for the involvement of a quinone-methide intermediate both in promoting carbon-oxygen bond cleavage of the acetate moiety and in serving as the actual substrate of the reduction reaction via NADPH-mediated hydride transfer.

**Table 2:** Relative activity of EGS variants

EGS variant	Relative activity* (%)
Wild type	100
Lys132Ala	0
Lys132Gln	0
Lys132Arg	71 (±6)
Tyr157Phe	32.5 (±4.4)
Tyr157Ala	21 (±3)
Ile261His	3.1 (±0.2)
Phe314Tyr	112 (±8)
Phe314Ala	54 (±5)
Phe314Tyr-Ala-Gln-Pro-Ser-Thr	115 (±11)
Δ311–314	34.9 (±0.7)

* The standard error of each activity measurement (derived from duplicate or triplicate determinations) is given in parentheses.

A prominent active-site residue is Lys132, which occurs in all PIP-family enzymes as well as most SDRs [17]. The structure of the EGS-NADP^+^-EMDF complex shows that the ε-amino group of Lys132 forms interactions with both the nicotinamide-ribose of NADP(H), and potentially, the substrate molecule (Figure 5A). The Lys132 interaction with the substrate is particularly intriguing, as it is not proximal to the site of hydride addition (as suggested in the case of IFR [7]), but instead involves the p-hydroxyl group via a bridging water molecule. Notably, a p-hydroxyl group is a distinguishing feature of the substrates of all PIP-family enzymes, and is a requirement for reduction by EGS [4]. For both PLR and IFR, alanine replacements of the lysine that is equivalent to EGS-Lys132 abolish enzyme activity [6], [7]. In EGS, Lys132Ala and Lys132Gln mutants are completely inactive, whereas the Lys132Arg mutant retains partial (71%) activity (Table 2). Crystallographic analyses confirmed that for the Ala132 and Gln132 mutants, both NADP^+^ and EMDF binding are little affected (Figure 5C), despite the loss of the binding interactions normally contributed by Lys132. These results therefore point to a catalytic role for Lys132. In particular, the involvement of a catalytic group acting at the p-hydroxyl group clearly argues for the formation of a quinone methide as a reaction intermediate as opposed to direct nucleophilic replacement by a NADPH derived hydride anion.

**Figure 6:**
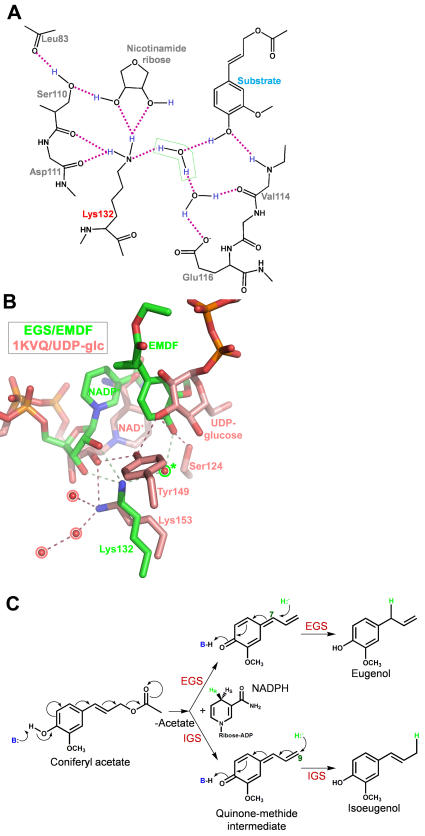
Hydrogen-bonding interactions in the EGS active-site and proposed reaction mechanism of EGS. (A) Hydrogen-bonding network involving the Lys132 side-chain amino group, the 4-hydroxyl group of EMDF, and the bridging water molecule. Inferred hydrogen-atom positions are shown in blue. Hydrogen-bond interactions are represented as magenta dashed lines. (B) Comparison of the hydrogen-bond interactions made by the catalytic lysine residue in EGS (Lys132) and the SDR UDP-galactose-4-epimerase (Lys153). The inset shows the coloring used for the carbon atoms of EMDF bound to EGS, and UDP-glucose bound to UDP-galactose-4-epimerase. Hydrogen bonds are drawn as thin dashed lines. Water molecules are drawn as red spheres. Those outlined in the pale red form part of a postulated proton-relay network in UDP-galactose-4-epimerase, whereas the presumed catalytic water molecule in EGS is outlined in green and marked with an asterisk. (C) Proposed reaction mechanism of EGS (and IGS) involving a quinone-methide intermediate. The catalytic base (B:) that promotes deprotonation of the 4-hydroxyl group of the substrate is the hydroxide ion that is activated by the side-chain amino group of Lys132. The loss of acetate generates the quinone-methide intermediate. The attack at C7 of this intermediate by the NADPH-derived hydride yields the allyl phenylpropene eugenol, whereas hydride attack at C9 presumably yields the iso-phenylpropene isoeugenol.

The formation of a quinone methide can be promoted by abstraction of the proton from the p-hydroxyl group of substrate. Detailed inspection of the hydrogen-bonding network involving the ε-amino group of Lys132 (Figure 6A) suggests that this group exists formally in the unprotonated–NH_2_ state and is the donor in hydrogen-bond interactions with the 2′-hydroxyl group of the nicotinamide-ribose and the backbone carbonyl oxygen of residue 110. With an available lone pair of electrons, the amino group can serve as the acceptor in a hydrogen bond with the bridging water molecule, and most importantly, thereby act as a general base. The water molecule (as a hydroxide ion) can in turn facilitate deprotonation of the substrate's p-hydroxyl group. Intruigingly, in the monoclinic structure of unliganded EGS-NADP^+^, a nitrate anion from the crystallization medium occupies the site of the bridging water molecule, and may mimic the hydroxide ion that develops during the catalytic reaction.

The loss in activity of EGS Lys132-mutants can be interpreted in terms of the proposed mechanistic model, in conjunction with results from structural analyses. In the EGS (Lys132Gln)-NADP^+^-EMDF complex, the Gln132 side chain retains an interaction with the nicotinamide ribose (also through an intervening water molecule), but is unable to form a direct or water-mediated interaction with the p-hydroxyl group of EMDF. Likewise, Ala132 obviously lacks a catalytic group capable of promoting deprotonation of the substrate's p-hydroxyl group, and therefore, the observed inactivity of the Lys132Ala and Lys132Gln mutants can be readily explained. The Lys132Arg mutant is partially active, and in this case, with the higher pK_a_ and greater length of the Arg side chain, the positively charged guanidinium moiety could possibly participate directly (i.e. without the requirement for an intervening water molecule) in lowering the pK_a_ of the substrate's p-hydroxyl group. Interestingly, preliminary structural analysis of the holo and EMDF-bound forms of the Lys132Arg mutant shows that the Arg132 guanidinium moiety is displaced slightly by the EMDF guaiacol ring (Figure 5D), thus diminishing the potential influence of the Arg132 on catalysis.

One difficulty with the proposed catalytic role for a lysine residue is the relatively high pK_a_ (normally ∼10.4 in solution) of the side-chain amino group, which would disfavor acquisition of the initial free-base state required for proton abstraction from the substrate. However, the pK_a_s of ionizable groups in proteins can be greatly influenced by the local structural environment, in particular, involvement in hydrogen-bonding networks and hydrophobic interactions. Such factors have been suggested to account for the catalytic-base activity of the lysine ε-amino group in a number of enzymes. A notable example is isochorismate synthase [18], in which a catalytic lysine is proposed to deprotonate and thereby activate a nucleophilic water molecule. Furthermore, from theoretical calculations on the conserved Lys-Tyr-Ser catalytic triad in an SDR-type alcohol dehydrogenase, the catalytically important lysine residue is suggested to exist in a partially unprotonated state, and in this state, participate in a proton-relay network that involves hydroxyl groups on the catalytic tyrosine and nicotinamide ribose [19]. This network functions ultimately to abstract a proton from the alcohol substrate. Intriguingly, although EGS lacks the catalytic tyrosine of the SDR enzyme, Lys132 in EGS corresponds exactly to the catalytic lysine of the SDRs, and the p-hydroxyl group of EGS-bound EMDF occurs at roughly the same position as the Tyr ζ-hydroxyl group of the catalytic tyrosine of SDRs (Figure 6B).

For the EGS-catalyzed reaction with the coniferyl acetate substrate, formation of the quinone-methide intermediate would be concomitant with displacement of an acetate ion (Figure 6C). In concert with proton abstraction from the p-hydroxyl group, EGS may therefore exploit an additional driving force for generation of the reaction intermediate—promoting the loss of the acetate. In particular, steric restrictions within the enzyme's active site (as discussed above) appear to disfavor the binding of an extended C1 substituent on the substrate. Furthermore, due to the predominantly non-polar character of the capping region of the EGS active site, only a single residue, Tyr157, is available for hydrogen-bond interactions with the polar oxygen atoms of the acetate moiety. In fact, the cluster of aromatic side-chains in this region of the active site (see Figure 5A) may provide a favorable environment for stabilizing the carbocationic character of C7 and C9 [20] within the extended quinone-methide. The proposed model for disfavoring binding of the acetate group is consistent with the diminished catalytic activity of mutants of EGS that carry smaller active-site capping residues (Tyr157Ala, Tyr157Phe, Phe314Ala, and Δ311–314; see Table 2).

Both the Tyr157Ala and Tyr157Phe mutants retain partial enzyme activity (21% and 33%, respectively; Table 2), and therefore, the hydrogen-bonding capacity of Tyr157 is apparently not essential for catalysis. The lack of a suitable proton donor within the active site of EGS to catalyze the carbon-oxygen bond cleavage may account for the requirement of EGS for an esterified substrate: coniferyl alcohol carries a much poorer leaving group (free hydroxide ion) than its acetylated form (the resonance-stabilized acetate ion) (see Figure 6C). The absence of a well-defined binding site for the acetate, underscored by the finding that soaking of EGS-NADP^+^ crystals with a high concentration (0.5 M) of sodium acetate yielded no ordered binding of acetate ion, may also contribute to the apparent irreversibility of the reaction that generates eugenol and acetate from coniferyl acetate.

In summary, a mechanistic scheme emerges in which binding of the coniferyl acetate substrate within the active site of EGS leads to deprotonation of the p-hydroxy group (PUSH) coupled with expulsion of acetate ion from the C1 substituent (PULL). The resultant extended quinone methide intermediate serves as a hydride acceptor at C7 to yield the product eugenol. For NAD(P)H-mediated reduction reactions, the acceptance of hydride by a substrate is typically accompanied by the acquistion of a proton at an adjacent atom, to maintain charge neutrality. As discussed above, EGS lacks an appropriately positioned proton donor near the site of hydride addition (C7); protonation instead occurs at the p-hydroxyl group, in concert with rearrangement of the double-bond system of the quinone methide and re-aromatization of the phenyl ring. Furthermore, the reduction of a double bond within a quinone methide intermediate more closely resembles a typical reaction catalyzed by a nicotinamide-cofactor enzyme, and indeed the reaction catalyzed by the PIP-family member IFR [10].

### Determinants of the regioselectivity of the EGS-catalyzed reduction reaction

In the structure of the EGS-NADP^+^-EMDF complex, the C4 atom of the cofactor's nicotinamide ring, which serves as the donor of the pro-R hydride, is directly apposed to the C7 atom of the inhibitor's side group (with an interatomic separation of 3.5 Å). Such a relative positioning of the nicotinamide ring and the side group of coniferyl acetate would appear to be ideal for hydride attack on the substrate C7-carbon and the consequent production of the expected allylphenylpropene, eugenol. In contrast, the production of the isophenylpropene, isoeugenol, would seemingly require hydride attack on the C9 carbon of the side group. Thus, a binding mode that more appropriately apposes the coniferyl-acetate C9 and nicotinamide C4 atoms would provide a possible means for conferring isoeugenol production from by the related enzyme, IGS from petunia. Further structural and mutagenic studies and analyses of additional, newly discovered IGS-type enzymes (Koeduka et al., submitted) are underway to probe the enzymic determinants of the regioselectivity of the EGS/IGS catalyzed reactions.

### Modeling of NADP(H) and substrate binding in other IFR-like proteins

The EGS residues that are involved in interactions with the NADP(H) cofactor (see above) are highly conserved in the other IFR-like enzymes, and thus these enzymes can be expected to maintain a cofactor-binding site very similar to that observed in EGS. Most of these other enzymes have also been characterized as A-type reductases. On the basis of the holo-EGS structures, the NADP(H) cofactor can be readily modeled into the apo-structures of the other IFR-like enzymes, although small, accommodating adjustments to the surrounding protein are necessary.

The substrates of the PLR, PCBER and IFR enzymes all possess a phenyl ring with a C4-hydroxyl group, and the expected sites of hydride addition occur near the C7 atom of the substrate. For these enzymes, substrate binding can be modeled on the basis of the positioning of the guaiacol moiety of EMDF bound to EGS. As expected, in all cases, the substrate C7-atom is positioned very close to the C4 atom of the cofactor's nicotinamide ring. The substrate-binding pocket in general appears less enclosed in the other IFR-like proteins than in EGS, consistent perhaps with the larger size of the C1 substituents of the cognate substrates. The more open binding pockets are due primarily to the absence of the C-terminal tail that occurs in EGS, as well as the substitution of smaller residues within the active-site capping region.

Moreover, the substrates for PLR and PCBER contain a cyclic-ether linkage adjacent to the site of hydride addition, and will therefore generate a transient alkoxide intermediate upon carbon-oxygen bond cleavage and ring opening. These enzymes may employ two means to promote protonation of the alkoxide intermediate: a potential proton donor or hydrogen-bonding group within the active site (His271 in PLR; Ser263 and Ser267 in PCBER), and a more open binding site that may allow access to the intermediate by bulk water.

## Materials and Methods

### Protein expression and purification

A DNA fragment encoding the entire amino-acid sequence (residues 1–314) of *Ocimum basilicum* EGS1 [4] was inserted between the NcoI and BamH1 sites of the expression vector pHIS8, which, under the control of a T7 promoter, yields the target protein fused to an N-terminal octahistidine tag [21]. For heterologous over-expression of the EGS protein, the plasmid pHIS8(EGS) was transformed into the expression host *E. coli* BL21(DE3) (Novagen). *E. coli* cultures in TB medium were grown at 37°C to an optical density (600 nm) of 1.5, induced with 1 mM isopropyl-β-D-thiogalactoside, and allowed to grow for an additional 6 hrs at 20°C. Bacterial cells were harvested by centrifugation, resuspended in lysis buffer (50 mM TrisHCl, pH 8.0; 0.5 M NaCl; 20 mM imidazole; 1% v/v Tween20; 10% v/v glycerol; and 20 mM 2-mercaptoethanol), and lysed by sonication. The EGS protein was isolated from the *E. coli* lysate by affinity chromatography with nickel-nitrilotriacetic-acid coupled agarose (Qiagen), and eluted with lysis buffer supplemented with 0.25 M imidazole. The partially purified EGS protein was treated with thrombin for cleavage of the octahistidine tag, and then further purified by gel-exclusion chromatography using a Superdex 200 HR26/60 column (Pharmacia Biosystems).

### Site-directed mutagenesis of *O. basilicum* EGS

Site-directed mutants of the EGS gene were created in the plasmid pHIS8(EGS) with the PCR method [22]. The DNA sequences of the mutant constructs were confirmed by sequencing of the entire EGS insert in both the forward and reverse directions.

### Chemoenzymatic synthesis of coniferyl acetate ((E)-4-hydroxy-3-methoxycinnamylacetate)

**Figure 7:**
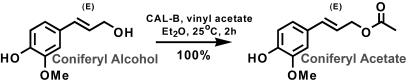
Chemoenzymatic synthesis of coniferyl acetate. Coniferyl acetate was obtained as a colorless oil with a yield of nearly 100%.

Coniferyl alcohol (180.2 mg, 1.0 mmol), *Candida antarctica* lipase B (CAL-B, 25 mg, Aldrich, ≥10,000 U/g), vinyl acetate (401 µL, 5.0 mmol), and dry diethyl ether (Et_2_O, 50 mL, 0.2 M) were stirred in a 125-mL Erlenmeyer flask at room temperature for 2 h (Figure 7). The reaction mixture was filtered through glass wool and concentrated *in vacuo*. The reaction yielded quantitatively the desired product (222 mg colorless oil). ^1^H NMR (500 MHz, CDCl3) δ 6.91 (m, 3H), 6.59 (d, *J* = 15.92 Hz, 1H), 6.15 (dt, *J* = 6.62, 15.92 Hz, 1H), 4.72 (d, *J* = 6.62 Hz, 2H), 3.92 (s, 3H), 2.11 (s, 3H); ^13^C NMR (125 MHz, CDCl3) δ 171.2, 146.8, 146.1, 134.7, 129.0, 121.0, 120.9, 114.7, 108.6, 65.5, 56.1, 21.3. LCMS [M-H]^-^ calculated for C_12_H_13_O_4_: 221.08, found: 221.2.

### Chemical synthesis of (7S,8S)-ethyl (7,8-methylene)-dihydroferulate (EMDF)

**Figure 8:**
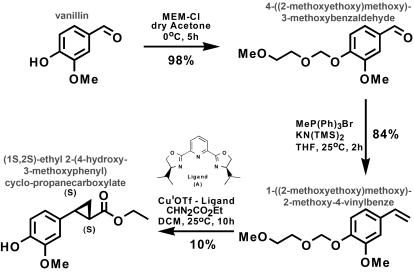
Chemical synthesis of (7S,8S)-ethyl (7,8-methylene)-dihydroferulate (EMDF). EMDF was obtained with an overall yield of 10%.

The compound (7S,8S)-ethyl (7,8-methylene)-dihydroferulate (EMDF, (1S,2S)-ethyl 2-(4-hydroxy-3-methoxyphenyl) cyclo-propanecarboxylate) was synthesized in three steps (Figure 8).

*4-((2-methoxyethoxy)methoxy)-3-methoxy benzaldehyde (step 1).* K_2_CO_3_ (1.38 g, 10.0 mmol, 1.0 eq.) was added to solution of vanillin (1.52 g, 10.0 mmol, 1.0 eq.) in dry acetone (70 mL, 0.15 M), and the mixture was stirred under argon at 0°C for 30 min. Then MeOCH_2_CH_2_OCH_2_Cl (MEM-Cl, 1.49 g, 12.0 mmol, 1.2 eq.) was added drop wise, and the mixture was stirred an additional 5 h. The mixture was then concentrated *in vacuo* to ∼20 mL, combined with 20 mL of water, and extracted with diethyl ether (3×25 mL). The organic layers were pooled, washed with brine, dried with MgSO_4_, filtered and concentrated *in vacuo*. The resulting yellow oil (2.38 g, 98% yield) was used without further purification. ^1^H NMR (500 MHz, CDCl_3_) δ 9.86 (s, 1H), 7.43 (s, 1H), 7.42 (d, *J* = 8.5 Hz, 2H), 7.32 (d, *J* = 8.5 Hz, 2H), 5.41 (s, 2H), 3.93 (s, 3H), 3.86 (t, *J* = 4.7 Hz, 2H), 3.54 (t, *J* = 4.7 Hz, 2H), 3.35 (s, 3H); ^13^C NMR (125 MHz, CDCl_3_) δ 191.0, 152.0, 150.0, 131.1, 126.5, 114.8, 109.4, 94.0, 71.4, 68.2, 59.0, 56.0; LCMS [M+Na]^+^ calculated for C_12_H_16_O_5_Na: 263.09, found: 263.3.

*1-((2-methoxyethoxy)methoxy)-2-methoxy-4-vinylbenzene (step 2).* To a suspension of methyltriphenylphosphonium bromide (MeP(Ph)_3_Br, 3.57 g, 10.0 mmol, 2.0 eq.) in anhydrous tetrahydrofuran (THF, 30 mL, 0.33 M), under argon at room temperature, was added potassium bis-trimethylsilylamide (KN(TMS)_2_, 4.5 mL, 9.0 mmol, 1.8 eq., 0.5 M in toluene). After 30 min, a solution containing 4-((2-methoxyethoxy)methoxy)-3-methoxy benzaldehyde (1.20 g, 5.0 mmol, 1.0 eq.) was added to the yellow colored ylide solution via cannula. The reaction was completed within 30–60 min (as monitored by TLC, hexanes/ethyl acetate, 7∶3). The reaction mixture was quenched with saturated NH_4_Cl and extracted with diethyl ether (3×100 mL). The organic layers were combined, washed with water (2×100 mL), brine (2×100 mL), dried over Na_2_SO_4_, filtered, concentrated *in vacuo*, and subjected to Dry Column Vacuum Chromatography (90% hexanes/ethyl acetate) to afford a colorless oil (2.01 g, 84% yield). ^1^H NMR (500 MHz, CDCl_3_) δ 7.15 (d, *J* = 8.3 Hz, 1H), 6.96 (s, 1H), 6.93 (d, *J* = 8.3 Hz, 1H), 6.65 (dd, *J* = 10.9, 17.5 Hz, 1H), 5.63 (d, *J* = 17.4 Hz, 1H), 5.32 (s, 2H), 5.17 (d, *J* = 10.9 Hz, 1H), 3.90 (s, 3H), 3.87 (t, *J* = 4.7 Hz, 2H), 3.56 (t, *J* = 4.7 Hz, 2H), 3.37 (s, 3H); ^13^C NMR (125 MHz, CDCl_3_) δ 149.7, 146.4, 136.4, 132.3, 119.4, 116.2, 112.4, 109.1, 94.4, 71.5, 67.8, 59.0, 55.8; LCMS [M+Na]^+^ calculated for C_13_H_18_O_4_Na: 261.1, found: 260.9.

*(1S,2S)-ethyl 2-(4-hydroxy-3-methoxyphenyl) cyclo-propanecarboxylate (step 3).* To a solution of copper-ligand complex prepared from Cu^I^OTf (toluene)_0.5 _(129.3 mg, 0.25 mmol, 2.0 mol%) and ligand **(A)** (75.3 mg, 0.25 mmol, 2.0 mol%) in 50 mL of dry dichloromethane (DCM) was added 1-((2-methoxyethoxy)methoxy)-2-methoxy-4-vinylbenzene (1.0 g, 4.2 mmol, 1.0 eq.). Ethyl diazoacetate (CHN_2_CO_2_Et, 485.8 µL, 4.62 mmol, 1.1 eq.) was dissolved in 0.5 mL dry dichloromethane and added to the stirring reaction mixture by slow addition via syringe pump over 10 h. The crude reaction mixture was filtered through a plug of silica gel and concentrated *in vacuo*. ^1^H NMR and LCMS showed a complex product mixture consisting of 80% conversion to cyclopropane products with a *trans* to *cis* ratio of 2.5∶1. Chiral HPLC analysis (Chiralcel-OD) determined 95% *ee* for the *trans* product. In addition to the MEM-protected trans- and cis-cyclopropane products, the crude reaction mixture contained the desired final product, the deprotected trans-cyclopropane, (1S,2S)-ethyl 2-(4-hydroxy-3-methoxyphenyl) cyclo-propanecarboxylate (EMDF), which was isolated by column chromatography (90% hexanes/ethyl acetate → 100% ethyl acetate) (99.2 mg as a colorless oil, 10% yield). Subsequent X-ray structure analysis of EMDF complexed with EGS confirmed the compound's absolute stereochemistry. ^1^H NMR (500 MHz, CDCl_3_) δ 6.82 (d, *J* = 8.2 Hz, 1H), 6.64 (s, 1H), 6.59 (d, *J* = 8.2 Hz, 1H), 5.56, ( br s, Ar-OH, 1H), 4.17 (q, *J* = 7.0 Hz, 2H), 3.87 (s, 3H), 2.47 (ddd, *J* = 4.6, 6.4, 9.6 Hz, 1H), 1.82 (ddd, *J* = 4.6, 5.6, 8.8 Hz, 1H), 1.54 (ddd, *J* = 4.8, 5.6, 9.2 Hz, 1H), 1.28 (t, *J* = 7.1 Hz, 3H)1.25 (ddd, buried, 1H); ^13^C NMR (125 MHz, CDCl_3_) δ 173.6, 146.5, 144.3, 131.9, 118.8, 114.3, 109.4, 60.7, 55.9, 26.1, 23.8, 16.7, 14.3; LCMS [M-H]^-^ calculated for C_13_H_15_O_4_Na: 235.1, found: 234.8.

### EGS enzyme assay

**Figure 9:**
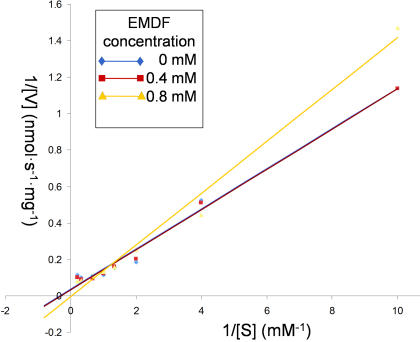
Double reciprocal plot for EGS activity in the presence and absence of EMDF. The plots illustrate the mixed nature of the EMDF EGS inhibitor. Ki was estimated from nonlinear fitting to a modified Michaelis-Menten equation.

EGS enzyme activity was measured by gas chromatography/mass spectrometry as described previously [4]. The assay mixture (total volume 0.15 mL) contained 0.05 M MES-KOH (pH 6.5), 1 mM NADPH, 1 mM coniferyl acetate, and 2 µg of EGS. Reaction mixtures were incubated at 25°C for 15 min followed by extraction with 1 mL of hexane. For determination of the specific activities of crude preparations of EGS, enzyme concentrations were assessed from western blots with an EGS antibody. For detailed kinetic analyses, substrate concentrations ranged from 0.1 to 5.0 mM, and for EMDF-inhibition determinations, the inhibitor concentrations used were 0, 0.4 and 0.8 mM (Figure 9).

### Crystallization of basil EGS

Wild-type EGS from basil (*Ocimum basilicum*) in complex with NADP^+^ was crystallized at 4°C from buffered solutions of protein mixed with polyethylene glycol (PEG) and a salt. The typical crystallization solutions employed were 0.1 M sodium succinate (pH 5.5), 5 mM NADP^+^ (Sigma Aldrich), 0.3 M KCl, 2 mM dithiothreitol and 21% (w/v) PEG 3350; or 0.1 M MOPSO (pH 6.5-7.0), 5 mM NADP^+^, 0.3 M KNO_3_, 2 mM dithiothreitol and 28% (w/v) PEG monomethylether 5000. These conditions yielded a number of distinct (but related) crystal forms. Morphologically, all of the crystal forms grew as thin plates. The two most commonly observed forms were monoclinic (space group *P*2_1_, with unit-cell parameters a = 53.8, b = 85.9, c = 76.2 Å, β = 107.3°) and orthorhombic (space group *P*2_1_2_1_2_1_, with unit-cell dimensions a = 79.3, b = 85.9, c = 99.2 Å), and both diffracted X-rays to high resolution (typically 1.6 to 2.0 Å). Similar crystallization conditions were also employed for wild-type EGS complexed with NADPH (the reduced form of the cofactor) or without added cofactor (apo-EGS), and for Lys132-mutant EGS proteins complexed with NADP^+^. (It should be noted that the apo-EGS protein likely contained a small amount of nicotinamide cofactor incorporated during expression in *E. coli*.) Micro-seeding was useful for promoting crystal growth of the mutant forms of EGS. Ternary complexes of EGS, NADP^+^ and the EGS inhibitor EMDF were obtained by soaking EGS/NADP^+^ crystals in crystallization solution supplemented with 5–10 mM EMDF.

### X-ray diffraction data

Crystals were transferred briefly to a cryoprotectant solution (consisting of reservoir solution supplemented with 17–20% v/v glycerol) prior to immersion in liquid nitrogen. X-ray diffraction data were measured from frozen crystals at beamlines 8.2.1 and 8.2.2 of the Advanced Light Source (Lawrence Berkeley National Laboratory) on ADSC Quantum 210 or 315 CCD detectors. Diffraction intensities were indexed, integrated and scaled with the programs XDS and XSCALE [23] or Mosflm [24] and Scala [25].

### X-ray structure determination of EGS

Initial crystallographic phases were determined for the orthorhombic crystal form of the EGS/NADP^+^ complex through molecular-replacement (MR) with the program Molrep [26]. A homology model for EGS was constructed with the program Modeller [27] based on the structure of phenylcoumaran benzylic ether reductase (PCBER, PDB entry 1QYC [6]). The program ARP/wARP was used for automated rebuilding of the initial structural model against a two-fold, non-crystallographic-symmetry averaged map. Subsequent structural refinement used the program CNS [28]. Xfit [29] was used for graphical map inspection and manual rebuilding of the atomic model. Programs from the CCP4 [30] suite were employed for all other crystallographic calculations. Structural depictions were generated with the program Pymol (Delano Scientific, San Carlos, CA).
